# Establishment and evaluation of a predictive model for length of hospital stay after total knee arthroplasty: A single-center retrospective study in China

**DOI:** 10.3389/fsurg.2023.1102371

**Published:** 2023-04-06

**Authors:** Bo Zhu, Dejun Zhang, Maocheng Sang, Long Zhao, Chaoqun Wang, Yunqiang Xu

**Affiliations:** Department of Orthopedics, Tianjin Medical University General Hospital, Tianjin, China

**Keywords:** total knee arthroplasty (TKA), length of hospital stay (LOS), nomogram, prediction model, payment method

## Abstract

**Background:**

Total knee arthroplasty (TKA) is the ultimate option for end-stage osteoarthritis, and the demand of this procedure are increasing every year. The length of hospital stay (LOS) greatly affects the overall cost of joint arthroplasty. The purpose of this study was to develop and validate a predictive model using perioperative data to estimate the risk of prolonged LOS in patients undergoing TKA.

**Methods:**

Data for 694 patients after TKA collected retrospectively in our department were analyzed by logistic regression models. Multi-variable logistic regression modeling with forward stepwise elimination was used to determine reduced parameters and establish a prediction model. The discrimination efficacy, calibration efficacy, and clinical utility of the prediction model were evaluated.

**Results:**

Eight independent predictors were identified: non-medical insurance payment, Charlson Comorbidity Index (CCI) ≥ 3, body mass index (BMI) > 25.2, surgery on Monday, age > 67.5, postoperative complications, blood transfusion, and operation time > 120.5 min had a higher probability of hospitalization for ≥6 days. The model had good discrimination [area under the curve (AUC), 0.802 95% CI, 0.754–0.850]] and good calibration (*p* = 0.929). A decision curve analysis proved that the nomogram was clinically effective.

**Conclusion:**

This study identified risk factors for prolonged hospital stay in patients after TKA. It is important to recognize all the factors that affect hospital LOS to try to maximize the use of medical resources, optimize hospital LOS and ultimately optimize the care of our patients.

## Introduction

Osteoarthritis is a major cause of pain and disability in patients, leading to increased socioeconomic costs worldwide (1). With the increase in the aging populations and the rising rates of obesity in developed countries, there will be a rapid multiplication in the rate of incidence of knee osteoarthritis (2). In 2012, the prevalence of symptomatic knee osteoarthritis was estimated to be around 8.1% in China (3). Total knee arthroplasty (TKA) is an elective procedure to manage intractable pain and functional limitations caused due to end-stage knee arthritis (4–6). An increase in the number of TKA procedures, along with rising healthcare costs, necessitates efforts to reduce the cost of treatment without compromising its quality.

The total cost of these procedures is primarily determined by the cost of joint implants, hospital stay, and operating room time (7). The cost of prostheses accounts for 60% of the TKA costs in China (8). However, the total proportion of prostheses cost in a TKA procedure is lower in some Western countries. An Italy-based study reported that the cost of a prosthesis for primary TKA was less than 50% (9), whereas a US-based study reported a TKA prosthesis cost about 30% of the total cost (10). The implementation of the centralized procurement policy in China is the reason behind the proportion becoming lower. As the proportion of hospitalization costs will further increase, reducing the length of stay (LOS), after TKA is essential to ease the economic burden on the patients as well as the healthcare system. The LOS following TKA has decreased substantially in recent years, which is somewhat related to the introduction of enhanced recovery protocols emphasizing multimodal analgesia and rapid mobilization (11, 12). Day-case surgeries have become quite challenging in joint arthroplasties. However, the implementation of “fast-track” pathways in Chinese hospitals has been infrequent, and the LOS after TKA has not been extensively researched (13, 14). This study aimed to develop a prediction model to identify factors that can predict a longer LOS following TKA.

## Patients and methods

### Study population

This study was a retrospective cohort of 694 consecutive patients who underwent a primary TKA at Tianjin Medical University General Hospital from July 2018 to April 2022. Data was collected retrospectively from electronic medical record system and individuals with incomplete demographic information were excluded. Patients with simultaneous bilateral surgery or revision were excluded. Ethics approval was obtained from Tianjin Medical University General Hospital. (approval number: IRB2023-WZ-008).

Based on the previous literature, the following 17 variables of interest were included: Age, Sex, body mass index (BMI), Residence, Primary diagnosis, Payment method, History of joint replacement, Preoperative range of motion (ROM), Charlson Comorbidity Index (CCI), American Society of Anesthesiologists (ASA) classification, Preoperative hemoglobin, Preoperative serum albumin, Surgeon, Day of operation, Operation time, Transfusion and Complications. Comorbidities were weighted according to the Charlson score (15). The comorbidities were summed and patient awarded a final comorbidity score. Postoperative complications included neurological complications, cardiac complications, infections, postoperative hypotension, and skin allergies.

All patients undergoing TKA are managed using a coordinated clinical pathway to ensure standardized medical, pharmacological pain management, and rehabilitation care. As a routine practice, 100 ml of cefazolin or cefuroxime was given intravenously 30 min before surgery, and clindamycin was administered to patients with a positive skin test. The surgical approach adopts the standard parapatellar retinaculum approach. All patients underwent cemented posterior-stabilized total knee arthroplasty, performed by 3 different surgical teams. Postoperatively, flurbinofen axetil or dezocine was used to relieve pain, and low molecular weight heparin was used to prevent thrombosis. Active and passive muscle exercises were started on the 1st postoperative day until the patient was discharged. Patients who underwent TKA were allowed to be discharged when they met the following discharge criteria: (1) Normal body temperature and no fever; (2) Able to walk independently with a walker; (3) No complications and/or comorbidities requiring hospitalization; (4) Postoperative ROM reaches 90° or significantly better than before operation to meet daily activities. Length of stay was defined as time between hospital admission and discharge. Those patients with a LOS greater than 75th percentile for all length of stays were defined as patients with prolonged length of stay (pLOS) (16, 17).

### Statistical analysis

The descriptive analysis presented continuous variables as mean (standard deviation) or categorical variables as counts (percentage). The Mann-Whitney U test was used to determine the significance of differences observed between the two groups for continuous variables, while the Chi-Square test was used for categorical variables. Fisher's Exact test was used for cases where the sample size was insufficient to perform a Chi-Square test (fewer than 5 observations in a cell). Receiver operating characteristic (ROC) curve analysis was used to determine the optimal cut-offs of continuous variables. The odds ratios (ORs) with their 95% confidence intervals (CIs) were derived.

Multi-variate logistic regression analysis with forward selection were applied to screen the risk factors for pLOS. Receiver operating characteristic (ROC) curve analysis was used to determine the optimal cut-offs of continuous variables. The logistic regression model was established by including the risk factors, with pLOS as the prediction. The optimal model selection was performed by applying a forward stepwise selection procedure through a likelihood-ratio test with Akaike information criterion (AIC). A nomogram was constructed based on the risk factors selected by the multi-variate logistic regression test. The nomogram's prediction accuracy was evaluated by C statistic, calibration curve, decision curve (DCA). The C statistic estimates the probability of concordance between predicted and observed outcomes in rank order and is equivalent to the area under the receiver operating curve (18). A C statistic of 0.5 indicates the absence of discrimination, whereas a C statistic of 1.0 indicates perfect separation of patients with different outcomes (19). The model was validated using bootstrapped resampling to quantify any overfitting. Statistical analysis was performed in R (version 4.2.1; R Development Core Team). A p value <0.05 (two tailed) was considered statistically significant.

## Results

### Patient characteristics

**Figure 1:**
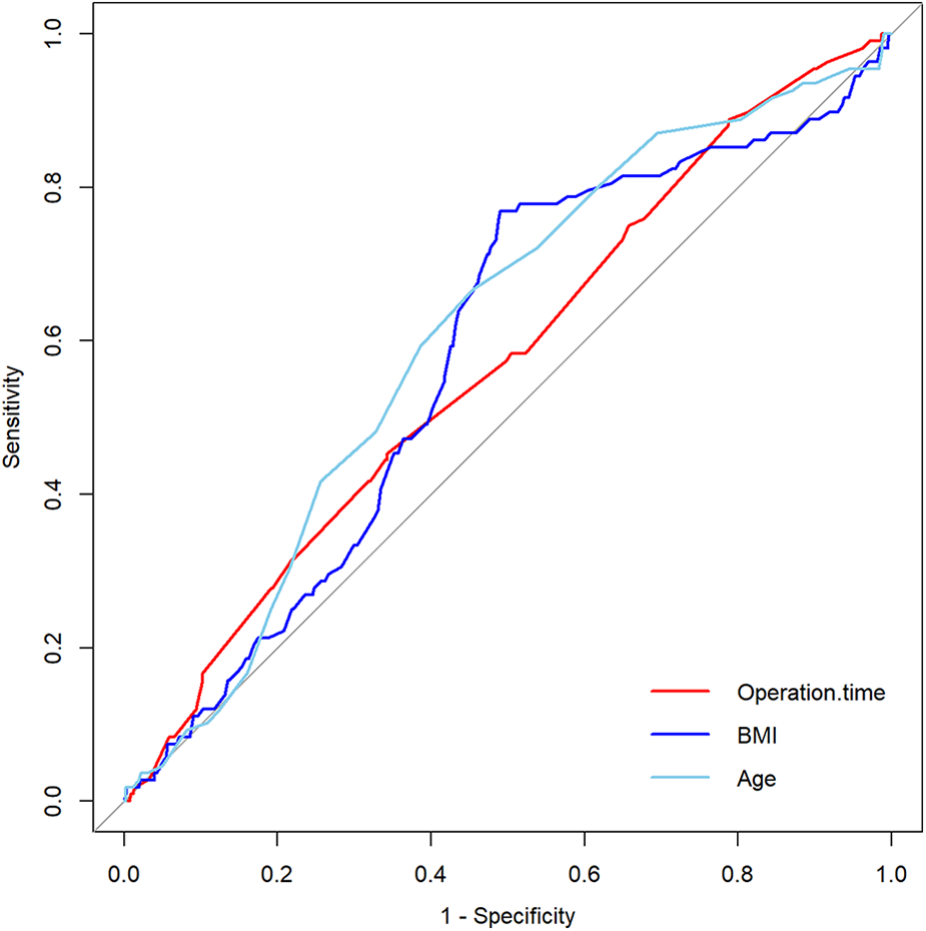
The ROC curve of continuous variables was investigated by using univariate analysis.

Table 1 compares demographic characteristics, comorbidities, surgery-related factors, and complications between patients with normal and prolonged hospital stays. The 75th percentile LOS for the overall sample was 6 days, with 108 out of 694 patients experiencing pLOS (>6 days). Patients with poor preoperative health had a higher likelihood of experiencing pLOS. Univariate analysis revealed that 22 patients had a CCI index ≥3, and 12 of them had pLOS (*P* < 0.001). A total of 20 patients developed complications after surgery, out of which 15 of them had pLOS. The top three complications included neurological (6 cases), cardiovascular (5 cases), and postoperative infection (5 cases). Continuous variables were dichotomized using the ROC curve (Figure 1 and Table 2). Patients with age >67.5, CCI > 3, BMI > 25.2 kg/m^2^, and operation time >120.5 min were defined as the cut-off values and were associated with a significantly higher incidence of pLOS.

**Table 1:** Demographics and clinical characteristics of patients by length of stay.

		Length of stay		
Characteristic	Combined	≤6 days	>6 days	*p*-value
Patients (*n*)	694	586 (84.4)	108 (15.6)	
Age ( > 67.5 yrs), *N* (%)	501 (72.2)	407 (58.7)	94 (13.5)	**<0.001**
Operation time ( > 120.5 min), *N* (%)	116.9 (24.3)	116.0 (24.5)	121.7 (22.9)	0.018
BMI( > 25.2 kg/m^2^), *N*(%)	371 (53.5)	288 (41.5)	83 (12.0)	**<0.001**
Mean(sd) Hb (g/L)	132.0 (14.1)	132.0 (14.2)	132.0 (13.0)	0.764
Mean(sd) ALB(g/L)	39.3 (3.5)	39.3 (3.4)	39.3 (3.6)	0.792
Gender, *N* (%)				0.509
Female	476 (68.6)	399 (57.5)	77 (11.1)	
Male	218 (31.4)	187 (26.9)	31 (4.5)	
Payment method, *N* (%)				**<0.001**
Medicaid	565 (81.4)	492 (70.9)	73 (10.5)	
Uninsured	129 (18.6)	94 (13.6)	35 (5.0)	
Residence, *N* (%)				0.921
Urban	299 (43.1)	252 (36.3)	47 (6.8)	
* *Rural	395 (56.9)	334 (48.1)	61 (8.8)	
Primary diagnosis, *N* (%)				0.791
OA	659 (95.0)	557 (80.3)	102 (14.7)	
RA	35 (5.0)	29 (4.2)	6 (0.8)	
History of joint replacement (Yes, %)	118 (17.0)	18 (2.6)	100 (14.4)	0.857
CCI (≥3), *N* (%)	22 (3.2)	10 (1.4)	12 (1.7)	**<0.001**
Procedure day of week, *N* (%)				**<0.001**
Monday	28 (4.0)	17 (2.4)	11 (1.6)	
Other	666 (96.0)	569 (82.0)	97 (14.0)	
ROM (<90°), *N* (%)	47 (6.8)	38 (5.5)	9 (1.3)	0.482
ASA score, *N* (%)				0.255
I/II	482 (69.5)	412 (59.4)	70 (10.1)	
III/IV	212 (30.6)	174 (25.1)	38 (5.5)	
Surgeon				**<0.001**
1	396 (57.1)	324 (46.7)	72 (10.4)	
2	259 (37.3)	236 (34.0)	23 (3.3)	
3	39 (5.6)	26 (3.7)	13 (1.9)	
Transfusion (Yes, %)	9 (1.3)	2 (0.3)	7(1.0)	**<0.001**
Complications, *N* (%)	20(2.9)	5(0.7)	15(2.2)	**<0.001**

BMI, body mass index; Hb, hemoglobin; ALB, albumin; OA, osteoarthritis; RA, rheumatoid arthritis; CCI, Charlson Comorbidity Index; ASA, American Society of Anesthesiologist.

The bold values highlight meaningful *p*-values with statistical significance.

**Table 2:** The AUC of continuous variables were investigated by using univariate analysis.

	AUC	95%CI	*P*	Cutoff	Sen	Spe
Operation time	0.571	0.514∼0.629	0.009	>120.5	0.454	0.657
BMI	0.582	0.525∼0.638	0.004	> 25.2	0.769	0.510
Age	0.610	0.555∼0.665	0.000	>67.5	0.667	0.546

### Multi-variate logistic regression analysis

**Figure 2:**
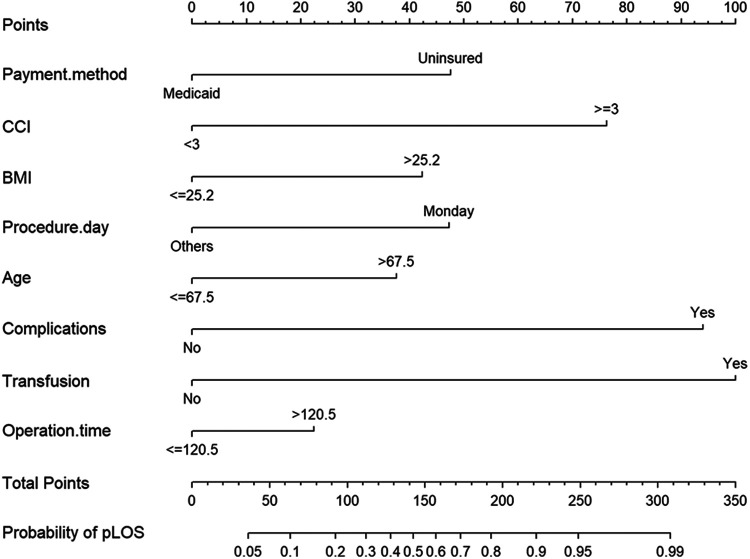
Nomogram of multivariable prediction model for LOS after TKA.

Patient factors in Table 1 were used in a multivariate regression analysis. Patients with age ≥ 67.5, BMI ≥ 25.2 kg/m^2^, CCI ≥ 3, non-Medicare payments, surgery on Monday, operation time ≥120.5 min, and postoperative complications had significantly higher odds of pLOS. A multivariate logistic regression analysis was used to establish a prediction model (Table 3), which was presented as a nomogram (Figure 2).

**Table 3:** Results of the multi-variate logistic regression analysis.

Predictor	OR	95%CI	*P*
Uninsured payment	3.74	2.14–6.54	<0.0001
CCI ≥ 3	8.29	2.87–23.89	<0.0001
BMI > 25.2	3.24	1.92–5.44	<0.0001
Surgery on Monday	3.71	1.43–9.64	0.0071
Age > 67.5	2.84	1.72–4.67	<0.0001
Complications	13.53	4.50–40.73	<0.0001
Transfusion	15.99	2.86–89.21	0.0016
Operation time > 120.5	1.86	1.14–3.04	0.0130

CCI, Charlson Comorbidity Index; BMI, body mass index.

### Model performance

**Figure 3:**
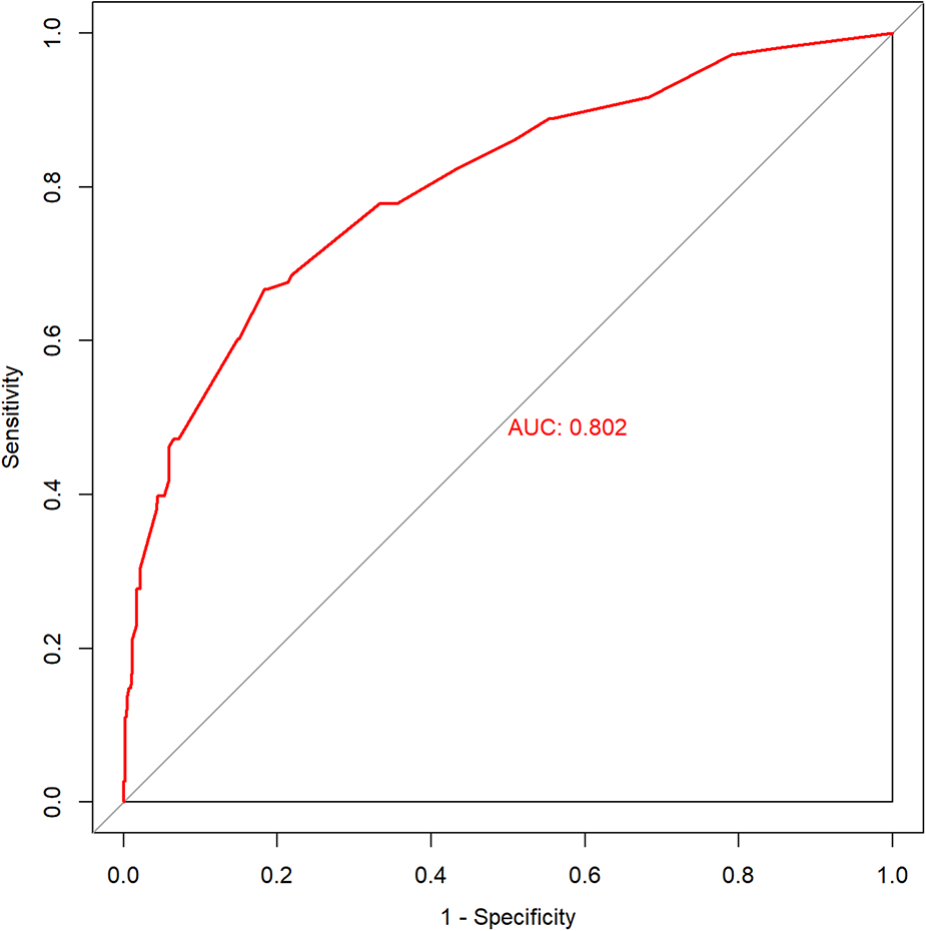
The ROC curve analysis of the nomogram.

**Figure 4:**
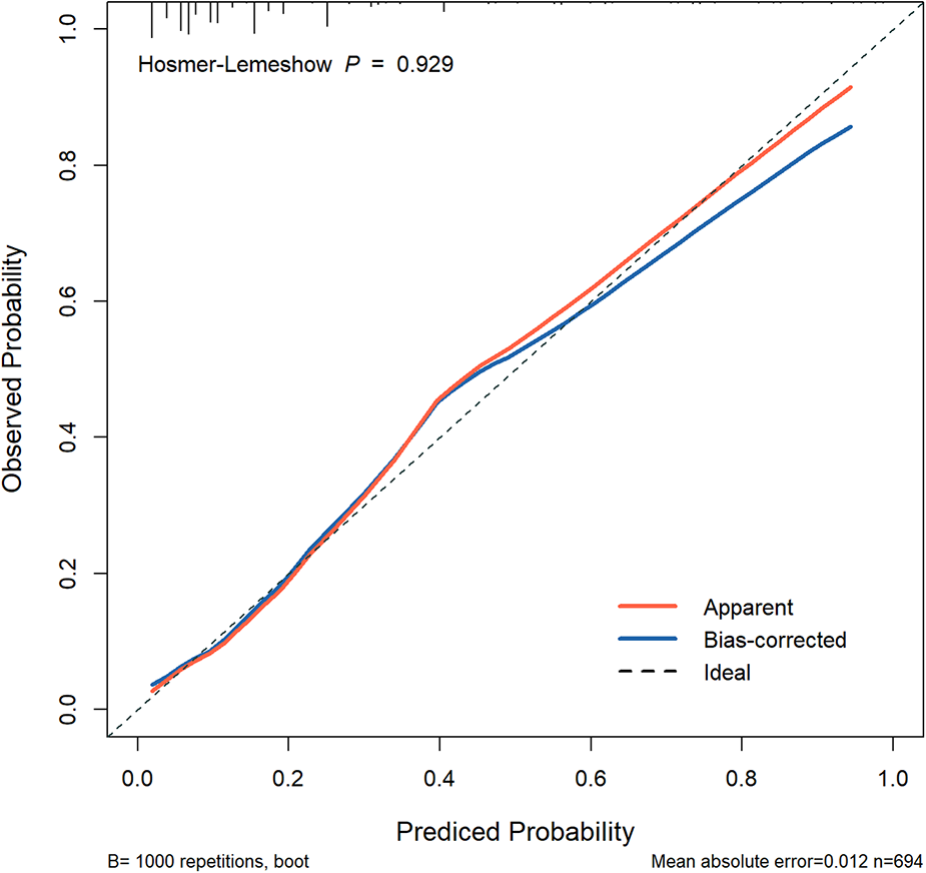
The calibration curve analysis of the nomogram.

**Figure 5:**
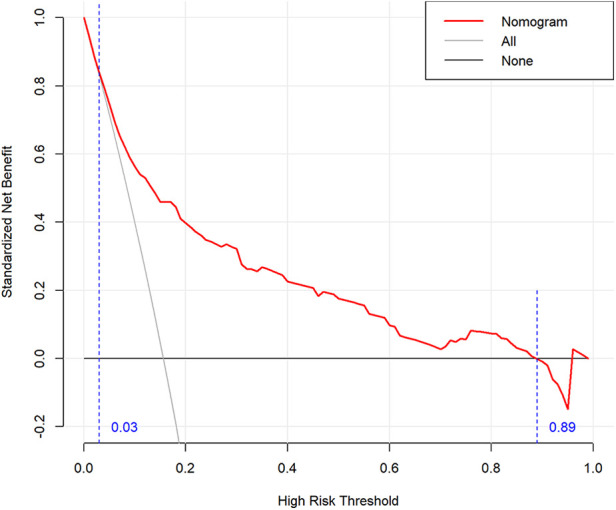
Decision analysis curve of a nomogram predicting LOS.

The predictive model was used to draw the ROC curve, and the area under the curve was calculated to be 0.802 (Figure 3). Internal validation with bootstrapping showed that the C-statistic of the risk score was 80.2% (95% CI:75.4–85.0) (the mean absolute error was 0.012). A good agreement between nomogram prediction and actual observation was observed from the calibration curve for the probability of pLOS (Figure 4). The Hosmer–Lemeshow test did not present a significant difference (*p* = 0.929), demonstrating a good fit without deviation. The DCA curve showed that the nomogram could receive a higher net benefit than either the “all had pLOS” or “none had pLOS” scheme because the threshold probability was within the range of 0.03 to 0.89 (Figure 5). Therefore, this nomogram has good applicability in predicting the probability of pLOS following TKA in clinical practice.

## Discussion

LOS is critical in hospital resource allocation due to the current scenario of cost-conscious in healthcare (20). Advances in surgical techniques, such as the use of a minimally invasive approach (21), multiple pain management modalities (11), and early mobilization after surgery, have aided in reducing LOS in hospitalized patients (22). Moreover, the upcoming payment model for diagnosis-related groups will further stimulate shorter LOSs.

A well-developed clinical nomogram can be used for the prediction of individual outcomes, benefitting both patients and clinicians. A nomogram has been used for risk assessment of adverse events following total joint arthroplasty in prior orthopedic studies (23–25). As Han et al. said in the article (14), the realization of the predictive system for LOS is the starting point for the promotion of fast-track arthroplasty in China. In this study, the risk factors associated with pLOS after TKA were identified. To the best of our knowledge, this is the first nomogram-based study to predict LOS after TKA in China.

Certain preoperative comorbidities have been linked to postoperative complications, which can lead to longer hospital stays. The CCI is a comorbidity index that can assess comorbidity burden (20). Patients with CCI ≥ 3 are more likely to have pLOS, which is consistent with previous research (26, 27). Individualized management based on the CCI scores during the preoperative assessment can help reduce LOS. While the effect of BMI on LOS after TKA remains controversial (27–29), our study found that a BMI > 25.2 increases the risk of LOS > 6 days. The duration of operation was also found to be strongly associated with longer LOSs. However, no association was found between the ASA score and hospitalization.

Advanced age was found to be a significant predictor of prolonged LOS, both individually and collectively (5). Smith et al. also observed a similar dose–response effect with an increase in LOS by 10%–13% for each decade of age (28). Similar findings were found in this study, highlighting the association between advanced age and LOS. As patients age, they typically suffer from an increasing number of comorbidities and declining physical function (30), resulting in a poorer ability to cope with surgical injury, which, in turn, affects mobilization and rehabilitation and prolongs LOS.

Since 2017, medical insurance payment methods based on single-disease payment have been implemented in China (31). The purpose of the single-disease payment model is to reduce the cost of medical services and improve the efficiency of medical insurance usage. This study found that uninsured patients were more likely to have pLOS. In this environment, medical insurance patients pay according to the payment standard, and the hospital will bear the excess expenses. Conversely, non-Medicare-paid patients do not have to worry about overpayment, and they tend to stay in the hospital longer to meet daily activities, leading to prolonged hospital stays. Medicaid patients were more likely to have ≥4 hospital LOS than Medicare patients (32). Medicaid patients are often individuals having low-income with complex social service needs and may have more complications that require more attention and management, leading to longer hospital stays. In addition, no prior reports on whether the payment method affects the LOS in China were found. Although Song et al. included the payment type in the statistical analysis in their study (15), the results revealed that the LOS of patients with different payment methods (Medicare vs. Self-pay) was not statistically significant. Variations in regions and populations may help account for differences in findings among the studies.

One interesting finding of this study is that patients who underwent surgery on Mondays had a higher risk of pLOS > 6 days than those who underwent surgery on other days. Procedures such as TKA have a fixed clinical pathway, with some patients scheduled for surgeries on Mondays and meeting discharge criteria by the weekend. However, patients may choose to delay discharge until the beginning of the following week due to various reasons, such as the inability to settle on weekends and the postponement of postoperative imaging review, which may increase their LOS. Previous studies have also reported similar findings (33, 34). Postoperative complications were identified as a significant predictor of pLOS, which is consistent with previous research (32, 35, 36). Unlike the study by Song et al. (13), this study does not exclude patients with complications. All patients with complications were discharged after their conditions improved or recovered, and the medical costs associated with their treatments cannot be overlooked. The presence of such patients also hindered the implementation of the “fast-track” protocols. Adverse events during hospitalization prolong the LOS for patients and increase the overall cost, and place additional burdens on patients and the health care system. Therefore, further research and efforts are required to reduce the incidence of complications in patients undergoing TKA.

Previous studies have identified blood management as an important factor affecting LOS. However, only a limited number of studies have explored the relationship between blood transfusion and LOS. Husted et al. have identified allogeneic blood transfusion as the most important predictor of discharge around the third day of admission (34). Monsef et al. also found that allogeneic blood transfusion was the most critical factor in prolonging the LOS (7). The present study's multivariate regression analysis showed that perioperative blood transfusion was significantly associated with extended hospital stays. Optimizing perioperative management measures such as intraoperative tranexamic acid and the use of autologous blood can reduce surgical blood loss and the risk of blood transfusions, thereby decreasing LOS. This study did not find any effect of preoperative hemoglobin levels on the LOS in patients undergoing TKA.

This study has several limitations and unanswered questions. Firstly, this is a retrospective study conducted in a single center, which may result in the presence of selection bias. Furthermore, preoperative knee scores were not routinely collected, which could have overlooked the impact of disease severity on the LOS. Secondly, this study included patients with primary TKA carried out between July 2018 and April 2022, and factors such as improvements in surgical techniques and changes in clinical practice over time were not accounted for, which could have affected the predictions. In addition, only the impact of postoperative complications on the length of hospital stay of patients as a whole was analyzed. Further research should focus on examining the impact of different complications on the LOS for patients as well as the risk factors associated with these complications.

In terms of methodology, this study assessed the applicability of the model through discrimination, calibration, and clinical applicability. Internal validation was performed to avoid over-interpretation of the data, although possible over-fitting due to the selection of variables and thresholds could not be eliminated. Biases resulting from the combination and accuracy in different patient populations could not be assessed. Future studies could utilize datasets from other hospitals to validate the model and provide opportunities for model improvement.

## Conclusions

To the best of our knowledge, this is the first study in China to establish a nomogram for predicting factors of pLOS after TKA. The model demonstrated good performance in identification, calibration, and decision curve analysis. The multivariate regression analysis revealed that patients with non-medical insurance, CCI ≥ 3, BMI  >  25.2, surgery on Monday, age > 67.5 years, postoperative complications, blood transfusion, and operation time > 120.5 min had a higher probability of hospitalization for ≥6 days. Early identification of factors that increase LOS and optimization of perioperative protocols for TKA patients may help reduce the risk of pLOS, thus alleviating the financial burden on patients and the healthcare system.

## Data Availability

The original contributions presented in the study are included in the article/Supplementary Material, further inquiries can be directed to the corresponding author/s.
